# Outcome-sensitive multiple imputation: a simulation study

**DOI:** 10.1186/s12874-016-0281-5

**Published:** 2017-01-09

**Authors:** Evangelos Kontopantelis, Ian R. White, Matthew Sperrin, Iain Buchan

**Affiliations:** 1 The Farr Institute for Health Informatics Research, University of Manchester, Vaughan House, Manchester, M13 9GB UK; 2 NIHR School for Primary Care Research, Centre for Primary Care, Institute of Population Health, University of Manchester, Manchester, UK; 3 MRC Biostatistics Unit, Cambridge Institute of Public Health, Cambridge, UK

**Keywords:** Multiple imputation, Imputed outcome, Missing data, Missingness

## Abstract

**Background:**

Multiple imputation is frequently used to deal with missing data in healthcare research. Although it is known that the outcome should be included in the imputation model when imputing missing covariate values, it is not known whether it should be imputed. Similarly no clear recommendations exist on: the utility of incorporating a secondary outcome, if available, in the imputation model; the level of protection offered when data are missing not-at-random; the implications of the dataset size and missingness levels.

**Methods:**

We used realistic assumptions to generate thousands of datasets across a broad spectrum of contexts: three mechanisms of missingness (completely at random; at random; not at random); varying extents of missingness (20–80% missing data); and different sample sizes (1,000 or 10,000 cases). For each context we quantified the performance of a complete case analysis and seven multiple imputation methods which deleted cases with missing outcome before imputation, after imputation or not at all; included or did not include the outcome in the imputation models; and included or did not include a secondary outcome in the imputation models. Methods were compared on mean absolute error, bias, coverage and power over 1,000 datasets for each scenario.

**Results:**

Overall, there was very little to separate multiple imputation methods which included the outcome in the imputation model. Even when missingness was quite extensive, all multiple imputation approaches performed well. Incorporating a secondary outcome, moderately correlated with the outcome of interest, made very little difference. The dataset size and the extent of missingness affected performance, as expected. Multiple imputation methods protected less well against missingness not at random, but did offer some protection.

**Conclusions:**

As long as the outcome is included in the imputation model, there are very small performance differences between the possible multiple imputation approaches: no outcome imputation, imputation or imputation and deletion. All informative covariates, even with very high levels of missingness, should be included in the multiple imputation model. Multiple imputation offers some protection against a simple missing not at random mechanism.

**Electronic supplementary material:**

The online version of this article (doi:10.1186/s12874-016-0281-5) contains supplementary material, which is available to authorized users.

## Background

Missing data is a common obstacle to observational health science and its pitfalls are well known [1]. To exclude study subjects with any missing covariate observations, so called complete case analysis, is at best of low statistical power and at worst provides biased estimates.

The complexity of the missing data problem, or obtaining accurate inferential estimates in the presence of missing data, depends on the nature of the mechanism by which data are missing [2]. The less problematic scenario occurs when the probability of an observable data point being missing (the missingness probability) does not depend on any observed or unobserved parameters, and this missingness mechanism is known as Missing Completely at Random (MCAR). However, MCAR mechanisms are considered rare in practice, especially for surveys [3]. More commonly, the missingness probability depends on observed variables, and hence it can be accounted for by the information contained in the dataset. This missing data mechanism has been labelled Missing at Random (MAR). Finally, the most challenging missingness mechanism occurs when the missingness probability depends on unobserved values, and called Missing Not at Random (MNAR). Using self-reporting of sexual activity we can explore MAR and MNAR examples. A MAR scenario would arise if girls are less likely than boys to report whether they are sexually active, but sexually active teenagers are no more likely to report than those non-active. However, if sexually active girls are less likely to report than non-active girls, this would be an MNAR scenario.

Although suboptimal approaches to imputation are still routinely used [4], multiple imputation has been accepted by methodologists as the most appropriate framework for dealing with MCAR and MAR mechanisms [5]. Multiple imputation can be described in three steps: (a) drawing the missing data from their posterior predictive distribution under a posited Bayesian model, across *N* datasets; (b) analysing each dataset separately with a chosen method, usually a regression model; and (c) pooling the estimates and their standard errors across the *N* analyses using Rubin’s rules [2], allowing for the use of the within-imputation and between-estimation variation components in the calculations. Multiple imputation has a largely Bayesian rationale but it also works well in frequentist applications by providing nominal coverage levels and unbiased point estimates [2]. When the analysis model (Step 2) is Bayesian the resulting framework is fully Bayesian; alternatively, frequentist maximum-likelihood estimates can be used in the model to draw missing values [6, 7]. An interesting trait of multiple imputation is that it performs better at imputing missing predictors when outcome information is included in the models [8]. Although standard applications of multiple imputation do not deal with MNAR mechanisms, they can offer some protection against them [9]. Furthermore, multiple imputation can accommodate MNAR scenarios flexibly and is thus well-suited to sensitivity analyses [10].

This paper tackles five outstanding issues about multiple imputation. von Hippel argued that researchers should impute values for the outcome, but exclude cases with imputed outcomes when fitting the substantive model [11]. It is unclear when this is the best strategy compared with not imputing the outcome on the one hand, and using imputed outcomes in the analysis on the other hand.

Another question of practical interest is how much missingness should be considered manageable within a multiple imputation framework, i.e. is the performance of multiple imputation consistent as missingness increases and is there a level above which performance deteriorates to such an extent that it makes the method and data of little practical use? Although this has been answered for multiple imputation that does not impute the outcome, which was found to perform consistently across all missingness levels [12], the question remains for outcome imputations.

Also unknown is the role of multiple correlated outcomes in the imputation models. It is not uncommon for studies to collect information on two or more correlated outcomes. However, these outcomes are often analysed through separate multiple imputation models, which do not utilise the association of the outcome of interest with a second (or more) available outcome. Would the inclusion of a second outcome lead to an improved multiple imputation model?

In addition, as far as we know, the level of protection, if any, offered by current multiple imputation methods (i.e. methods that assume MAR) against MNAR mechanisms has not been quantified within a simulations framework. Although there are technical challenges and numerous assumptions when developing such a framework, a high level of protection would make researchers more confident when reporting results from analyses with missing data—especially since MNAR mechanisms cannot be identified without additional external data or prior knowledge.

Finally, the size of the investigated dataset could be an important parameter and the performance of multiple imputation has largely been assessed in small or moderate datasets [8, 13], mainly for computational reasons. We have chosen scenarios of 1000 cases, which would be relevant to small and moderate studies, and more or less in agreement with previous investigations. However, it is now common to analyse data from many thousands or even millions of people, for example using Electronic Health Records (EHRs), and for this reason we also analysed scenarios of 10,000 cases.

In this paper we address all of the questions above using simulations. We simulate a wide range of scenarios which are not uncommon in observational studies with databases of routinely collected data, where hundreds of variables may be available and often have varying levels of missingness [14]. Although our motivation stems from our experiences with observational data, our findings are also relevant to clinical trials data, which are usually less variable (e.g. levels of missingness across variables are uniform, when someone is lost to follow-up).

## Methods

Obtaining algebraic answers to the questions we have posed is challenging due to: the large number of parameters often involved; and the asymptotic estimation approaches commonly employed. Arguably the most reasonable approach is to use realistically simulated data, where the true associations between predictors and the outcome are known, and can be used reliably to quantify method performance. The processes described below were repeated 1,000 times, to obtain different datasets under the specified parameters and to analyse them.

### Data generation

**Fig. 1:**
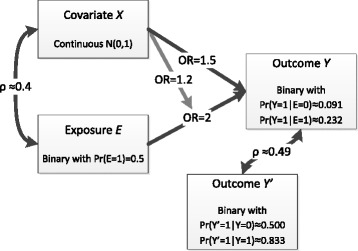
Data structure

We assumed two dataset sizes of 1,000 and 10,000 patients for which we originally had complete information on a primary binary outcome *Y*, a secondary binary outcome *Y*’, a binary exposure variable *E* and a continuous covariate *X* confounding the relationship between exposure and outcomes. The whole process was implemented in Stata v14.1 [15], and the code is provided in Online Additional file 1. We used the *drawnorm* command to draw observations from multivariate normal distributions, allowing for a ≈ 0.4 Pearson’s correlation between *X* and *E*. Covariate *X* had mean 0 and variance 1, while we set Pr (*E* = 1) = 0.5. Outcome *Y*’ was generated last, correlated with primary outcome *Y* (tetrachoric *ρ* ≈ 0.49), but independent of *X* and *E* given *Y*. Writing *π* = *p*(*Y* = 1|*X*, *E*), we assumed the logistic regression model logit(*π*) = *β*_0_ + *β*_1_ *E* + *β*_2_ *X* + *β*_3_ *E* ⋅ *X* with parameters: exp (*β*_0_) ≈ 0.091, exp (*β*_1_) = 2, exp (*β*_2_) = 1.5 and exp (*β*_3_) = 1.2. These parameters lead to conditional probabilities for the outcome of Pr (*Y* = 1|*E* = 0) = 0.091 and Pr (*Y* = 1|*E* = 1) = 0.232. Weaker associations exist between *Y*’ and *E*, *Y*’ and *X*, *Y* ^’^ and *X* * *E*, but are not of interest. The data structure is displayed in Fig. 1.

### Missingness mechanisms

We implemented three missing data mechanisms (MCAR, MAR and MNAR) and four levels of overall missingness for each variable (20%, 40%, 60% and 80%). In each case, covariate *X* and outcomes *Y* and *Y* ^’^ all had the same level of missingness but information for exposure *E* was always complete. In the MCAR setting, values for *X*, *Y* and *Y*’ were independently set to be missing. In the MAR setting, the probability to be missing for each of *X*, *Y* or *Y*’ was independently set to be conditional on the exposure *E* with *OR* = 5. In other words, the odds of a missing value for *X*, *Y* or *Y*’ were five times as high in the presence of the exposure (i.e. when *E* = 1). In the final setting, a relatively simple MNAR scenario, the probabilities of missing data for *X*, *Y* or *Y*’ were conditional on the true values of *X*, *Y* or *Y*’ respectively, with *OR* = 5 used across each of the associations. Information for exposure *E* was always complete. However, the MNAR scenario is of course not exhaustive and alternative MNAR mechanisms could vary across exposure groups [16]. It should also be noted that although the missingness mechanism we modelled is rather extreme, it was a conscious decision to make it more likely to observe performance variability across models. We anticipate our findings to be relevant to weaker associations, where model performances are expected be less variable.

### Alternative data structures

We also considered two alternative structures, as sensitivity analyses, which we do not present in detail in this paper but the code for which is available from the authors. In the first sensitivity analysis we simulated a continuous rather than a binary outcome, and in the second sensitivity analysis we included a second covariate *X*’ to which we applied the same missingness mechanisms.

### Analysis

Across each missingness mechanism we followed the same seven logistic regression analyses, seven of which were multiple imputation approaches (Table 1). The first analysis (A) was the simplest, a complete cases analysis, with the sole purpose of providing a benchmark against which to compare the multiple imputation approaches. In the remaining seven analyses we used the *mi* family of commands in Stata, with *mi impute chained* for the imputation and *mi estimate* with a multiple logistic regression (*logit* command) for the analysis. Analyses B, C, D and E all ignored the secondary outcome *Y*’. In the second analysis (B), which is known to give biased estimates [11], we excluded cases where the outcome was missing and the outcome was not included in the imputation model. In the third analysis (C), we again dropped missing outcome cases but included the outcome in the imputation model for the missing covariate *X*. In the fourth analysis (D) we included the outcome in the imputation model and imputed its missing values as well as missing values for *X*. An alternative approach suggested by von Hippel [11] was our analysis E, which followed D and included the outcome in the multiple imputation model and imputing it, but deleted cases where the outcome was imputed. Analyses F and G, followed C and D respectively but also included the second outcome in the imputation models and imputed their missing values. Finally, analysis H followed the setup of D, except it did not include the covariate *X* in the multiple imputation or analysis models. This aimed to assess whether the covariate should be included, even when its missingness levels were very high.

**Table 1:** Analysis methods

A	complete case analysis (no multiple imputation [mi])
B	no outcome imputation, not included in mi model
C^a^	no outcome imputation, outcome imputed in mi model
D^a^	outcome imputed and included in mi model
E^a^	outcome imputed and included in mi model but then cases where it was imputed are deleted
F^a^	as in C but also including a second correlated outcome in the mi model
G^a^	as in D but also including a second correlated outcome in the mi model
H	as in D but the mi and analysis models do not include the covariate

^**a**^Main models of interest, other models provided for comparison purposes

The analysis approaches and the data setup were selected to fit our research questions. Comparing analysis models C and D, which are the commonly recommended best practice models, will provide information on whether imputing an incomplete outcome is preferable to excluding the relevant cases. Model E, which deletes cases where the outcome is imputed, will be assessed as a best practice alternative to C and D. Comparisons between C and F, as well as D and G will inform us whether the inclusion of a second outcome which is correlated to our outcome of interest leads to a better multiple imputation model. Comparing models that have included the covariate (e.g. C and D) with model H, across various levels of missingness, will answer whether it is preferable to exclude a covariate from a multiple imputation model when most of its values are missing. Simulating different missingness mechanisms will allow us to quantify the performance of multiple imputation approaches vs complete case analyses in the most problematic MNAR scenario, compared to the known protection it offers for the most common MCAR and MCAR scenarios [13]. Finally, repeating the analyses in datasets of different sizes will shed light to whether our conclusions are sample-size dependent or not.

### Performance measures

We aimed to measure the performance of all multiple imputation and analysis approaches with logistic regression, across the scenarios of missingness described above and over 1,000 iterations, in the estimation of the three true association of interest: *E* → ^*OR*=2^ *Y* (our main focus), *X* → ^*OR*=1.5^ *Y* and *X* * *E* → ^*OR*=1.2^ *Y*. There are numerous performance measures that can be used in simulation studies [17], but we considered mean absolute error, mean bias, coverage probability and power of the analyses in relation to the three parameters of interest to be adequate for our investigation. Mean absolute error was calculated as $\frac{1}{1000}{\displaystyle {\sum}_{i=1}^{1000}}\left| z-{\hat{z}}_i\right|$ where *z* is one of the three parameters of interest, expressed as a log-odds ratio. Analogously mean bias is the mean difference in the estimate to the true parameter, and was calculated as $\frac{1}{1000}{\displaystyle {\sum}_{i=1}^{1000}}\left( z-{\hat{z}}_i\right)$. Therefore, assuming the used exposure effect of log[2], a reported mean bias of 0.1 or −0.1 would mean that the returned estimate was on average ≈ log(1.81) or ≈ log(2.21), respectively. The coverage probability, is the proportion of 95% confidence intervals for the estimate that contain the true parameter across the 1,000 iterations. Theoretically this should be close to 95% but the bias introduced through the MAR and MNAR mechanisms can affect coverage levels. Finally, we calculated the power to detect that the parameter is different from zero by computing the proportion of the 1,000 95% confidence intervals for each parameter that did not include zero. However, power needs to be carefully interpreted in the presence of bias since bias will move the estimate closer or further away from the alternative hypothesis on which power is calculated, and in the latter case higher bias will lead to higher power. Nevertheless, provided bias is similar across the methods to compare, power can be used for comparisons, even if bias is not zero, and we felt it was an important metric that would complement the study.

## Results

**Fig. 2:**
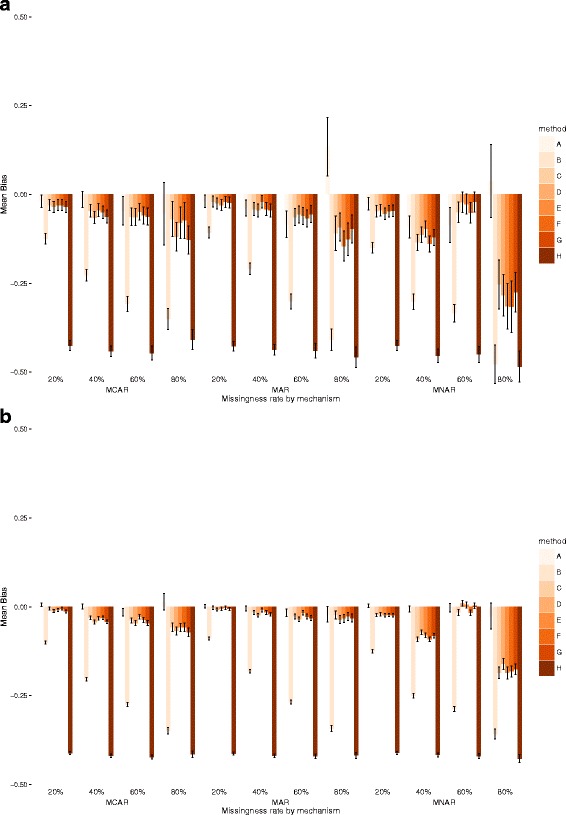
Mean Bias and 95% Confidence Intervals for exposure E in datasets of 1000 (top) and 10,000 observations (bottom)

**Fig. 3:**
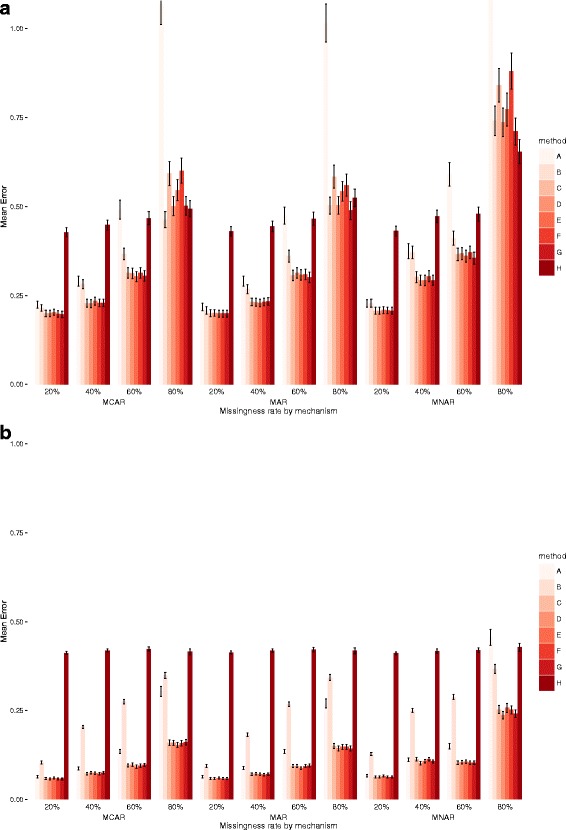
Mean absolute Error and 95% Confidence Intervals for exposure E in datasets of 1000 (top) and 10,000 observations (bottom)

**Fig. 4:**
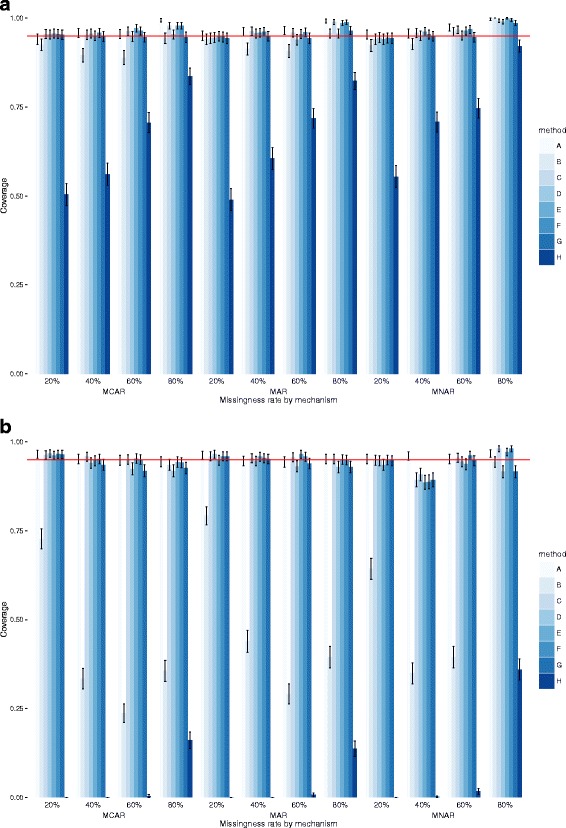
Coverage and 95% Confidence Intervals for exposure E in datasets of 1000 (top) and 10,000 observations (bottom)

**Fig. 5:**
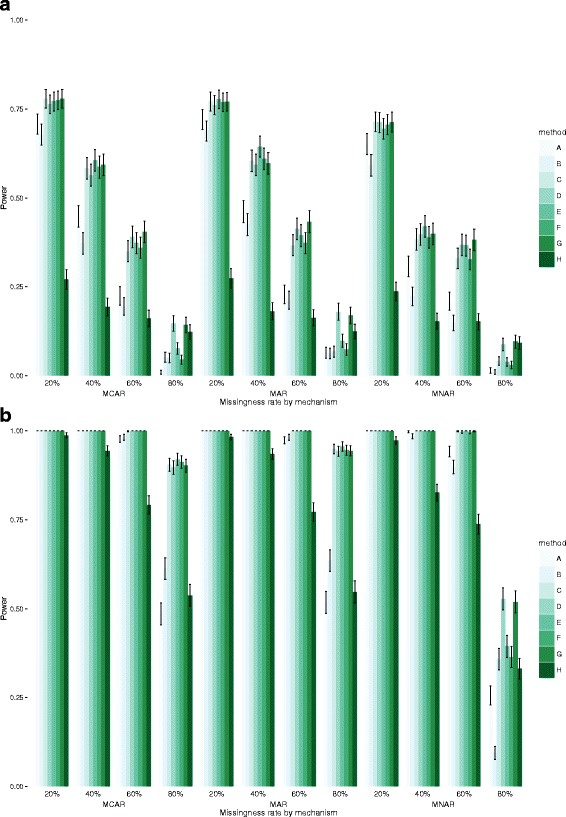
Power and 95% Confidence Intervals for exposure E in datasets of 1000 (top) and 10,000 observations (bottom)

Results for the coefficient of the exposure *β*_1_ are presented in Table 2 and Table 3 for datasets of 1,000 and 10,000 cases, respectively. Figures 2, 3, 4 and 5 present the exposure performance metrics with their respective error bars. Although all methods successfully converged for the larger datasets, there was some variation in the smaller datasets for very high levels of missingness (Online Additional file 2: Table S5). Results for the coefficients of the covariate and the exposure-covariate interaction are also presented in Online Additional file 2: Table S1, S2, S3 and S4).

**Table 2:** Performance results for exposure E, datasets of 1,000 observations^a^

		% miss	A	B	C^b^	D^b^	E^b^	F^b^	G^b^	H
MCAR	Mean bias^c^	20	−0.019	−0.107	−0.020	−0.025	−0.030	−0.021	−0.023	−0.427
		40	−0.038	−0.209	−0.041	−0.045	−0.020	−0.041	−0.045	−0.437
		60	−0.084	−0.301	−0.064	−0.056	−0.060	−0.067	−0.055	−0.440
		80	0.134	−0.409	−0.109	−0.092	−0.145	−0.126	−0.097	−0.458
	Mean error^c^	20	0.218	0.208	0.200	0.201	0.199	0.199	0.199	0.430
		40	0.290	0.268	0.232	0.231	0.229	0.232	0.233	0.444
		60	0.475	0.361	0.306	0.313	0.308	0.309	0.301	0.466
		80	1.016	0.503	0.584	0.503	0.543	0.560	0.489	0.525
	Coverage	20	0.950	0.941	0.943	0.945	0.949	0.947	0.943	0.489
		40	0.962	0.913	0.963	0.957	0.959	0.962	0.949	0.605
		60	0.965	0.907	0.959	0.939	0.955	0.960	0.943	0.718
		80	0.992	0.957	0.989	0.956	0.986	0.988	0.965	0.823
	Power	20	0.720	0.688	0.771	0.761	0.777	0.769	0.770	0.274
		40	0.462	0.425	0.604	0.593	0.644	0.610	0.597	0.181
		60	0.228	0.213	0.367	0.413	0.394	0.373	0.433	0.163
		80	0.065	0.062	0.068	0.180	0.098	0.074	0.169	0.124
MAR	Mean bias^c^	20	−0.019	−0.125	−0.030	−0.035	−0.030	−0.030	−0.035	−0.425
		40	−0.014	−0.227	−0.046	−0.065	−0.044	−0.050	−0.062	−0.441
		60	−0.046	−0.308	−0.063	−0.064	−0.049	−0.059	−0.062	−0.446
		80	−0.054	−0.350	−0.069	−0.119	−0.080	−0.073	−0.128	−0.408
	Mean error^c^	20	0.224	0.215	0.200	0.200	0.203	0.198	0.197	0.428
		40	0.290	0.282	0.228	0.227	0.234	0.229	0.229	0.448
		60	0.491	0.367	0.314	0.312	0.303	0.314	0.305	0.467
		80	1.070	0.463	0.593	0.501	0.546	0.601	0.502	0.494
	Coverage	20	0.941	0.925	0.955	0.954	0.956	0.955	0.953	0.504
		40	0.958	0.896	0.953	0.956	0.950	0.958	0.948	0.561
		60	0.955	0.889	0.963	0.948	0.971	0.964	0.946	0.706
		80	0.994	0.943	0.978	0.954	0.978	0.978	0.946	0.836
	Power	20	0.708	0.678	0.779	0.763	0.771	0.774	0.779	0.271
		40	0.448	0.372	0.583	0.564	0.606	0.587	0.593	0.193
		60	0.224	0.195	0.350	0.391	0.373	0.360	0.404	0.161
		80	0.010	0.052	0.050	0.147	0.077	0.045	0.143	0.123
MNAR	Mean bias^c^	20	−0.026	−0.150	−0.047	−0.044	−0.054	−0.047	−0.046	−0.425
		40	−0.092	−0.302	−0.135	−0.113	−0.097	−0.139	−0.121	−0.454
		60	−0.086	−0.334	−0.050	−0.022	−0.027	−0.052	−0.021	−0.450
		80	0.038	−0.478	−0.253	−0.283	−0.314	−0.316	−0.275	−0.484
	Mean error^c^	20	0.227	0.228	0.207	0.207	0.209	0.208	0.207	0.431
		40	0.375	0.371	0.302	0.293	0.292	0.304	0.293	0.472
		60	0.590	0.411	0.366	0.368	0.361	0.371	0.355	0.479
		80	1.283	0.741	0.841	0.737	0.773	0.881	0.711	0.654
	Coverage	20	0.954	0.923	0.940	0.945	0.941	0.944	0.943	0.554
		40	0.957	0.927	0.958	0.950	0.961	0.954	0.949	0.708
		60	0.973	0.962	0.967	0.951	0.964	0.968	0.946	0.746
		80	0.997	1.000	0.993	0.990	0.999	0.995	0.986	0.921
	Power	20	0.652	0.592	0.714	0.712	0.694	0.706	0.713	0.237
		40	0.307	0.223	0.383	0.398	0.420	0.389	0.399	0.153
		60	0.209	0.148	0.330	0.368	0.366	0.327	0.382	0.152
		80	0.015	0.010	0.041	0.088	0.039	0.030	0.096	0.092

^a^Analysis model A: complete case analysis (no multiple imputation [mi]); B: no outcome imputation, not included in mi model; C: no outcome imputation, outcome imputed in mi model; D: outcome imputed and included in mi model; E: outcome imputed and included in mi model but then observations where it was imputed are deleted; F as in C but also including a second correlated outcome in the mi model; G as in D but also including a second correlated outcome in the mi model; H as in D but the mi and analysis models do not include the covariate

^b^Main models of interest, other models provided for comparison purposes

^c^Reported on log-odds scale and based on a true effect of log [2]

**Table 3:** Performance results for exposure E, datasets of 10,000 observations^a^

		% miss	A	B	C^b^	D^b^	E^b^	F^b^	G^b^	H
MCAR	Mean bias^c^	20	0.001	−0.089	−0.003	−0.008	−0.006	−0.003	−0.006	−0.413
		40	−0.005	−0.181	−0.016	−0.024	−0.009	−0.016	−0.021	−0.419
		60	−0.017	−0.268	−0.027	−0.035	−0.017	−0.028	−0.032	−0.421
		80	−0.021	−0.343	−0.023	−0.036	−0.033	−0.027	−0.032	−0.418
	Mean error^c^	20	0.064	0.094	0.059	0.059	0.060	0.059	0.059	0.413
		40	0.088	0.182	0.071	0.072	0.071	0.070	0.071	0.419
		60	0.135	0.268	0.094	0.094	0.088	0.094	0.095	0.421
		80	0.270	0.343	0.150	0.144	0.148	0.148	0.143	0.418
	Coverage	20	0.962	0.792	0.959	0.965	0.948	0.959	0.959	0.000
		40	0.946	0.439	0.954	0.946	0.958	0.954	0.952	0.000
		60	0.943	0.291	0.956	0.931	0.965	0.958	0.939	0.007
		80	0.952	0.394	0.952	0.929	0.950	0.949	0.929	0.137
	Power	20	1.000	1.000	1.000	1.000	1.000	1.000	1.000	0.983
		40	1.000	1.000	1.000	1.000	1.000	1.000	1.000	0.935
		60	0.974	0.982	1.000	1.000	1.000	1.000	1.000	0.772
		80	0.518	0.635	0.949	0.943	0.956	0.946	0.944	0.547
MAR	Mean bias^c^	20	0.005	−0.101	−0.005	−0.012	−0.009	−0.005	−0.013	−0.412
		40	0.001	−0.204	−0.031	−0.043	−0.032	−0.031	−0.042	−0.419
		60	−0.015	−0.275	−0.038	−0.046	−0.028	−0.037	−0.046	−0.423
		80	0.014	−0.349	−0.058	−0.068	−0.058	−0.058	−0.072	−0.415
	Mean error^c^	20	0.064	0.104	0.059	0.058	0.060	0.058	0.058	0.412
		40	0.087	0.204	0.072	0.075	0.074	0.072	0.075	0.419
		60	0.135	0.275	0.096	0.098	0.092	0.095	0.097	0.423
		80	0.304	0.349	0.160	0.159	0.153	0.158	0.161	0.415
	Coverage	20	0.965	0.727	0.963	0.967	0.962	0.965	0.964	0.000
		40	0.952	0.334	0.958	0.940	0.947	0.951	0.935	0.000
		60	0.948	0.237	0.950	0.924	0.953	0.950	0.918	0.004
		80	0.944	0.356	0.935	0.918	0.943	0.941	0.926	0.161
	Power	20	1.000	1.000	1.000	1.000	1.000	1.000	1.000	0.987
		40	1.000	1.000	1.000	1.000	1.000	1.000	1.000	0.944
		60	0.977	0.982	0.999	1.000	1.000	1.000	1.000	0.792
		80	0.486	0.613	0.905	0.897	0.920	0.912	0.902	0.537
MNAR	Mean bias^c^	20	0.003	−0.125	−0.023	−0.021	−0.024	−0.023	−0.024	−0.411
		40	−0.006	−0.250	−0.091	−0.072	−0.080	−0.091	−0.081	−0.417
		60	−0.003	−0.288	−0.016	0.010	0.005	−0.017	0.003	−0.420
		80	−0.026	−0.358	−0.186	−0.161	−0.186	−0.182	−0.176	−0.427
	Mean error^c^	20	0.067	0.128	0.063	0.063	0.066	0.063	0.063	0.411
		40	0.112	0.250	0.113	0.102	0.107	0.113	0.107	0.417
		60	0.150	0.288	0.103	0.105	0.106	0.104	0.103	0.420
		80	0.456	0.367	0.253	0.237	0.258	0.252	0.241	0.428
	Coverage	20	0.952	0.643	0.948	0.947	0.935	0.949	0.947	0.000
		40	0.960	0.349	0.893	0.908	0.886	0.887	0.893	0.002
		60	0.952	0.394	0.955	0.944	0.937	0.961	0.947	0.017
		80	0.967	0.943	0.981	0.916	0.971	0.981	0.916	0.360
	Power	20	1.000	1.000	1.000	1.000	1.000	1.000	1.000	0.973
		40	0.998	0.985	1.000	1.000	1.000	1.000	1.000	0.826
		60	0.942	0.898	0.999	0.997	1.000	0.996	0.999	0.738
		80	0.256	0.094	0.358	0.527	0.394	0.364	0.519	0.331

^a^Analysis model A: complete case analysis (no multiple imputation [mi]); B: no outcome imputation, not included in mi model; C: no outcome imputation, outcome imputed in mi model; D: outcome imputed and included in mi model; E: outcome imputed and included in mi model but then observations where it was imputed are deleted; F as in C but also including a second correlated outcome in the mi model; G as in D but also including a second correlated outcome in the mi model; H as in D but the mi and analysis models do not include the covariate

^b^Main models of interest, other models provided for comparison purposes

^c^Reported on log-odds scale and based on a true effect of log [2]

### Mean bias

In smaller datasets and for MCAR and MAR data, levels of bias were low across most models, except in B (outcome not included in the imputation model) and H (covariate not included in the multiple imputation model). Complete case analysis (model A) was often the best performer, especially for low levels of missingness but results could only be obtained for a subsample of less problematic datasets, due to perfect prediction or non-convergence (Online Additional file 2: Table S5). Bias levels increased for MNAR data. In larger datasets bias levels were lower for all methods and complete case analysis appeared to be by the best performer with very low bias in every simulation scenario.

### Mean absolute error

In both smaller and larger datasets, the best performing models were: C (no outcome imputation, outcome included in mi model); D (outcome imputed and included in mi model); E (outcome imputed and included in mi model and then deleted); F (no outcome imputation, both outcomes included in mi model); and G (both outcomes included in mi model, outcome of interest imputed). For the smaller datasets, the models that imputed the outcome (D, E and G) generally performed only slightly better for very high levels of missingness in MCAR data, and for MAR data (and especially for higher rates of missingness). Error increased with increasing missingness but was not too dissimilar across the three missingness mechanisms. In the larger datasets, levels of mean absolute error were much lower and there was no benefit in using the second outcome, with models C, D and E performing the best, with variations in different settings. Overall, the best model was E but only slightly better than C and D.

### Coverage

Again, at both dataset sizes, models C, D, E, F and G performed best. There was very little to separate them, however, the models that imputed the outcome (D and G) tended to be closer to the nominal 95%. There were relatively small differences across missingness mechanisms and coverage levels were good in all scenarios, with the lowest rates amongst the five top performing models observed for D and G in MAR data and high missingness levels. Similarly, coverage rates were consistently high across all levels of missingness. In larger datasets, there was no benefit to using a second outcome, with models C, D and E equivalent in almost all missingness scenarios. Overall, differences between models C, D and E are small but E had better coverage in the larger datasets for extensive missingness.

### Power

Results for power were consistent with error and coverage, with models C, D, E, F and G again performing best. In the smaller datasets, there were small differences between these models, except for very high levels of missingness, and especially for MCAR and MAR mechanisms, where imputing the outcome (models D and G) returned higher power level, albeit still very low. However, for lower levels of missingness, model C performed well and, more often than not, slightly better than D. The nature of the missingness mechanism had some effect on power, with lower levels observed for MNAR data, especially as levels of missingness increased. As expected, the more data are missing the lower the power, and all models performed very poorly for high or very high levels of missingness (60% or above). In larger datasets, the picture did not change with models C, D, E, F and G being almost equivalent, but model D performed better for extensive missingness (60% or above) in MNAR data. Overall, E outperformed C is all settings and D for low and moderate levels of missingness, while D performed better for very high levels of missingness.

### Sensitivity analyses

Patterns of results in the two sets of sensitivity simulations broadly agreed with what we observed for the main simulations and further supported our findings. When analysing a continuous outcome, differences between multiple imputation models were again very small. Focusing on datasets of 1000 observations and the multiple imputation models of main interest (C to G), mean bias was very similar for all missingness mechanisms and levels. Mean bias was very close to zero for all MCAR and MAR settings, except for 80% levels of missingness. For MNAR data, mean bias was very close to zero for 20% missingness and linearly increased with missingness. Mean absolute error was again similar in these methods across all missingness mechanisms, with some variability being observed for 80% missingness and method G (outcome imputed and included in mi model, including secondary outcome) performing slightly better in those scenarios (Online Additional file 2: Figure S1). Coverage was similar in all scenarios, except for high levels of missingness where the outcome imputation models (G and especially D) slightly underperformed. However, that shortcoming was counterbalanced for model G by higher power in all scenarios except very low levels of missingness, where there was very little variation in performance (Online Additional file 2: Figure S2).

## Discussion

Our results indicate that in general, there are very small differences between models that impute the outcome compared with those that do not, when all else is equal and the outcome is included in the imputation model. However, in some contexts small differences emerge that should underpin recommendations as to the choice of model. The von Hippel approach [11], our model E, where the outcome is included in the imputation model and imputed but cases where the outcome is imputed are later dropped performed well. However, the differences between this approach and alternative models, where the outcome is not imputed or imputed and not dropped, were generally very small if any (error bars for all performance metrics overlapped substantially). Furthermore, the von Hippel approach was not consistently better in all scenarios. Another consideration is the presence of an “auxiliary” variable, a variable that in not part of the analysis model but is used in the multiple imputation to improve the prediction of missing values. If such a variable is associated with missingness in the outcome, model E is known to produce biased parameter estimates and should be avoided [18].

The level of missingness naturally affects the performance of the multiple imputation models, especially with regards to power (primarily) and error (secondarily). However, in agreement with Janssen et al. [12], we recommend using all available data even when missingness among covariates of interest is extensive. Multiple imputation models that exclude such covariates seem to perform much worse. For very high levels of missingness and moderately sized datasets we recommend the use of simulation-based platforms to estimate the power to detect effects [19]. Convergence was not an issue with any models when the datasets contained 10,000 observations, but it was a factor to consider in the 1,000 observations datasets as the level of missingness increased. Multiple imputation models that did not impute the outcome and were only modestly affected, while complete case analysis was severely affected.

The size of the datasets (1,000 or 10,000) did not substantially affect how the models ranked within each group. Interestingly, in the larger datasets, a complete case analysis approach was generally only slightly worse than the best performing multiple imputation models for low levels of missingness. Therefore, existing multiple imputation approaches may be less relevant to large health informatics databases than to randomised clinical trials.

Surprisingly the inclusion of a second outcome in the multiple imputation model, moderately correlated to the primary outcome, made very little difference to performance. Since for the imputation model there is no real distinction between predictors and outcomes, we would expect the inclusion of the secondary outcome to lead to improved performance. However, our findings could be explained by the associations between the predictors and the secondary outcome. In other words, the secondary outcome has little independent information to add to the model. A weaker association between predictors and secondary outcomes and a stronger correlation between outcomes would make the secondary outcome a useful addition to the multiple imputation model. However, we did observe slightly better performance for the model in the continuous outcome sensitivity analysis, for some scenarios, mainly in terms of power but also mean absolute error. Hence a more complete multiple imputation model that includes all outcomes is recommended.

Finally, although all models performed worse when data were MNAR, multiple imputation models can offer some protection, in terms of mean absolute error, even in this relatively extreme missingness scenario we simulated (OR = 5 for the missingness mechanism). Multiple imputation models outperformed complete case analyses in both smaller and larger datasets. However, the benefits of using multiple imputation methods were not as high for MNAR as for MCAR or MAR data, and were more obvious in the smaller datasets.

### Strengths and limitations

We have evaluated the performance of commonly used imputation approaches in realistic simulated data scenarios. Nevertheless, some limitations exist. First, although realistic, our simulated scenarios cannot be exhaustive and results may vary in alternative scenarios with different hypothesised associations between exposure, covariate and outcome and different distributions. However, we would expect the methods to perform similarly, at least relatively to each other, and our conclusions not to be affected—at least in MCAR settings. Our MAR settings made complete cases analysis (method A) unbiased because missingness depended only on exposure; if missingness of covariates had also depended on outcome then bias would have arisen in complete cases analysis. Regarding MNAR, we investigated common scenarios but there are many other possible mechanisms and our findings are not generalisable to them. In particular, our MNAR mechanism for *Y* was akin to case–control sampling and hence caused no bias. A different missing data mechanism that depended on both *Y* and *E* could cause large bias in the coefficient of *E*, especially if the association between missingness and *Y* differed across exposure groups [16]. Second, the precision obtained with simulations of 1,000 iterations is not ideal but the models we executed are complex and require considerable computational time. Third, a sample of 1,000 might seem too large if compared against trial data, but it was a necessity if we were to investigate very highc rates of missingness. Fourth, the substantive model was not entirely consistent with the imputation model because of the interaction term –we felt it was important to reflect this approach because it is often seen in practice. Fifth, we only considered one strength of association between the outcome *Y* and the secondary outcome *Y*’: although we modelled a rather strong association, probably stronger to what would be observed in practice in most cases, even stronger associations are likely to increase the value of including the secondary outcome in imputation models. Finally, the computational time led us to select our largest simulated dataset to include 10,000 cases. Unfortunately, this is not necessarily representative of a contemporary electronic health records dataset which can hold hundreds of thousands or millions of cases. However, even that limited size is very different to the size of a clinical trial, on which multiple imputation methods have been routinely evaluated in the past. Therefore, we argue that we manage to provide an incomplete view on the relevance of these methods in larger datasets.

## Conclusions

There was very little to separate the multiple imputation methods of interest. Although the method that imputes the outcome of interest and then removes observations where the outcome is imputed performed slightly better in some scenarios, especially for low and moderate levels of missingness, it was not always better and it is known to be biased in the presence of auxiliary variables. For very high levels of missingness, the higher power obtained when imputing the outcome (and not dropping observations) might make this approach somewhat more appealing. However, as long as the outcome is included in the imputation model, the choice of the multiple imputation approach makes no practical difference.

Important covariates need to be included in the imputation models even when their levels of missingness are very high. Although the use of secondary outcomes did not lead to substantially better models in our simulations, some improvements were observed in the sensitivity analysis, and we recommend their inclusion. Multiple imputation is the best approach across all missingness mechanisms and offers some protection in some simple missing not at random contexts.

## Additional files

Additional file 1: Simulation code file 1 of 4. Generate data and obtain true estimates (making sure the simulations work as they should before incorporating the missing data mechanisms). Simulation code file 2 of 4. Main data generation file across missingness mechanisms (1 of 2). Simulation code file 3 of 4. Main data generation file across missingness mechanisms (2 of 2). Simulation code file 4 of 4. Summarise the simulation results in a data file. (ZIP 10 kb)
Additional file 2: Supplementary file for “Outcome-sensitive Multiple Imputation: a Simulation Study”. Additional results for the covariate and the interaction term for the main analyses, but also all results from sensitivity analyses. (PDF 480 kb)

## Abbreviations

EHR: Electronic Health Record
MAR: Missing at random
MCAR: Missing completely at random
MNAR: Missing not at random
