# Supplementary material for: Outcome-sensitive multiple imputation: a simulation study
Source: BMC Med Res Methodol. 2017 Jan 9;17:2. doi: 10.1186/s12874-016-0281-5 (PMC5220613; doi:10.1186/s12874-016-0281-5)

# Supplementary file for “Outcome-sensitive Multiple Imputation: a Simulation Study”

**Table S1: Performance results for covariate X, datasets of 1,000 observations*†**

| | | % miss | A | B | C‡ | D‡ | E‡ | F‡ | G‡ |
|---|---|---|---|---|---|---|---|---|---|
| MCAR | Mean bias§ | 20 | -0.002 | 0.083 | -0.026 | -0.045 | -0.023 | -0.024 | -0.044 |
| | | 40 | -0.012 | 0.162 | -0.058 | -0.085 | -0.056 | -0.061 | -0.083 |
| | | 60 | -0.048 | 0.234 | -0.098 | -0.117 | -0.101 | -0.093 | -0.120 |
| | | 80 | -0.076 | 0.316 | -0.191 | -0.141 | -0.116 | -0.170 | -0.127 |
| | Mean error§ | 20 | 0.186 | 0.163 | 0.165 | 0.151 | 0.164 | 0.165 | 0.152 |
| | | 40 | 0.254 | 0.204 | 0.195 | 0.175 | 0.188 | 0.194 | 0.177 |
| | | 60 | 0.397 | 0.260 | 0.263 | 0.237 | 0.269 | 0.261 | 0.240 |
| | | 80 | 0.843 | 0.340 | 0.553 | 0.451 | 0.504 | 0.528 | 0.428 |
| | Coverage | 20 | 0.949 | 0.959 | 0.960 | 0.980 | 0.972 | 0.964 | 0.974 |
| | | 40 | 0.943 | 0.972 | 0.983 | 0.987 | 0.982 | 0.985 | 0.984 |
| | | 60 | 0.960 | 0.986 | 0.990 | 0.971 | 0.985 | 0.987 | 0.970 |
| | | 80 | 0.996 | 0.999 | 0.994 | 0.957 | 0.992 | 0.998 | 0.963 |
| | Power | 20 | 0.855 | 0.960 | 0.863 | 0.872 | 0.870 | 0.855 | 0.876 |
| | | 40 | 0.625 | 0.932 | 0.608 | 0.680 | 0.628 | 0.630 | 0.670 |
| | | 60 | 0.339 | 0.841 | 0.303 | 0.423 | 0.322 | 0.320 | 0.413 |
| | | 80 | 0.093 | 0.414 | 0.104 | 0.182 | 0.103 | 0.110 | 0.188 |
| MAR | Mean bias§ | 20 | 0.003 | 0.065 | -0.012 | -0.027 | -0.018 | -0.012 | -0.026 |
| | | 40 | -0.007 | 0.121 | -0.035 | -0.052 | -0.029 | -0.034 | -0.053 |
| | | 60 | -0.014 | 0.228 | -0.073 | -0.102 | -0.075 | -0.075 | -0.106 |
| | | 80 | -0.096 | 0.292 | -0.097 | -0.055 | -0.089 | -0.079 | -0.038 |
| | Mean error§ | 20 | 0.176 | 0.158 | 0.161 | 0.152 | 0.158 | 0.161 | 0.152 |
| | | 40 | 0.206 | 0.173 | 0.177 | 0.165 | 0.181 | 0.179 | 0.170 |
| | | 60 | 0.336 | 0.253 | 0.258 | 0.241 | 0.258 | 0.252 | 0.241 |
| | | 80 | 0.687 | 0.311 | 0.544 | 0.461 | 0.494 | 0.525 | 0.446 |
| | Coverage | 20 | 0.939 | 0.956 | 0.959 | 0.967 | 0.961 | 0.960 | 0.972 |
| | | 40 | 0.956 | 0.969 | 0.975 | 0.974 | 0.975 | 0.976 | 0.973 |
| | | 60 | 0.957 | 0.989 | 0.982 | 0.965 | 0.982 | 0.984 | 0.962 |
| | | 80 | 0.979 | 0.994 | 0.966 | 0.947 | 0.979 | 0.970 | 0.955 |
| | Power | 20 | 0.887 | 0.965 | 0.902 | 0.900 | 0.896 | 0.897 | 0.916 |
| | | 40 | 0.760 | 0.955 | 0.756 | 0.776 | 0.777 | 0.763 | 0.775 |
| | | 60 | 0.400 | 0.882 | 0.368 | 0.433 | 0.399 | 0.383 | 0.441 |
| | | 80 | 0.190 | 0.584 | 0.172 | 0.217 | 0.163 | 0.186 | 0.235 |
| MNAR | Mean bias§ | 20 | 0.002 | 0.079 | -0.021 | -0.040 | -0.029 | -0.020 | -0.041 |
| | | 40 | -0.008 | 0.152 | -0.060 | -0.097 | -0.058 | -0.056 | -0.096 |
| | | 60 | -0.033 | 0.236 | -0.109 | -0.151 | -0.122 | -0.109 | -0.158 |
| | | 80 | -0.024 | 0.281 | -0.054 | -0.065 | -0.068 | -0.057 | -0.043 |
| | Mean error§ | 20 | 0.191 | 0.171 | 0.171 | 0.158 | 0.168 | 0.170 | 0.156 |
| | | 40 | 0.334 | 0.238 | 0.258 | 0.232 | 0.246 | 0.260 | 0.232 |
| | | 60 | 0.426 | 0.271 | 0.305 | 0.302 | 0.311 | 0.313 | 0.302 |
| | | 80 | 0.893 | 0.441 | 0.819 | 0.765 | 0.764 | 0.835 | 0.680 |
| | Coverage | 20 | 0.936 | 0.966 | 0.957 | 0.967 | 0.974 | 0.960 | 0.976 |
| | | 40 | 0.940 | 0.986 | 0.981 | 0.974 | 0.985 | 0.978 | 0.973 |
| | | 60 | 0.964 | 0.994 | 0.989 | 0.950 | 0.983 | 0.980 | 0.954 |
| | | 80 | 0.992 | 1.000 | 0.993 | 0.970 | 0.995 | 0.995 | 0.973 |

| | | % miss | A | B | C‡ | D‡ | E‡ | F‡ | G‡ |
|---|---|---|---|---|---|---|---|---|---|
| | Power | 20 | 0.826 | 0.930 | 0.836 | 0.842 | 0.810 | 0.834 | 0.833 |
| | | 40 | 0.445 | 0.723 | 0.415 | 0.435 | 0.432 | 0.420 | 0.424 |
| | | 60 | 0.283 | 0.731 | 0.253 | 0.313 | 0.257 | 0.258 | 0.307 |
| | | 80 | 0.050 | 0.056 | 0.037 | 0.084 | 0.051 | 0.053 | 0.090 |

*Analysis model A: complete case analysis (no multiple imputation [mi]); B: no outcome imputation, not included in mi model; C: no outcome imputation, outcome imputed in mi model; D: outcome imputed and included in mi model; E: outcome imputed and included in mi model but then observations where it was imputed are deleted; F as in C but also including a second correlated outcome in the mi model; G as in D but also including a second correlated outcome in the mi model.

† Analysis model H does not include the covariate and hence not reported.

‡ Main models of interest, other models provided for comparison purposes.

§ Reported on log-odds scale and based on a true effect of log(1.5)

**Table S2: Performance results for interaction term E*X, datasets of 1,000 observations*†**

| | | % miss | A | B | C‡ | D‡ | E‡ | F‡ | G‡ |
|---|---|---|---|---|---|---|---|---|---|
| MCAR | Mean bias§ | 20 | -0.006 | 0.038 | 0.031 | 0.063 | 0.034 | 0.030 | 0.061 |
| | | 40 | -0.003 | 0.080 | 0.073 | 0.115 | 0.066 | 0.077 | 0.114 |
| | | 60 | 0.011 | 0.125 | 0.117 | 0.148 | 0.106 | 0.119 | 0.155 |
| | | 80 | -0.117 | 0.153 | 0.165 | 0.176 | 0.147 | 0.172 | 0.178 |
| | Mean error§ | 20 | 0.219 | 0.178 | 0.181 | 0.153 | 0.181 | 0.180 | 0.154 |
| | | 40 | 0.310 | 0.195 | 0.194 | 0.151 | 0.192 | 0.199 | 0.154 |
| | | 60 | 0.488 | 0.214 | 0.217 | 0.158 | 0.217 | 0.216 | 0.163 |
| | | 80 | 1.120 | 0.249 | 0.288 | 0.177 | 0.266 | 0.278 | 0.180 |
| | Coverage | 20 | 0.942 | 0.977 | 0.979 | 0.991 | 0.977 | 0.979 | 0.991 |
| | | 40 | 0.931 | 0.993 | 0.992 | 0.997 | 0.992 | 0.993 | 0.996 |
| | | 60 | 0.963 | 1.000 | 1.000 | 1.000 | 1.000 | 0.998 | 0.998 |
| | | 80 | 0.996 | 1.000 | 1.000 | 1.000 | 1.000 | 1.000 | 1.000 |
| | Power | 20 | 0.733 | 0.822 | 0.812 | 0.902 | 0.829 | 0.813 | 0.908 |
| | | 40 | 0.452 | 0.682 | 0.658 | 0.939 | 0.650 | 0.660 | 0.937 |
| | | 60 | 0.238 | 0.436 | 0.388 | 0.945 | 0.363 | 0.381 | 0.956 |
| | | 80 | 0.032 | 0.066 | 0.027 | 0.850 | 0.036 | 0.036 | 0.857 |
| MAR | Mean bias§ | 20 | -0.003 | 0.094 | 0.034 | 0.061 | 0.037 | 0.034 | 0.061 |
| | | 40 | -0.024 | 0.175 | 0.063 | 0.112 | 0.072 | 0.067 | 0.110 |
| | | 60 | -0.060 | 0.148 | 0.098 | 0.146 | 0.106 | 0.096 | 0.149 |
| | | 80 | -0.078 | 0.221 | 0.141 | 0.178 | 0.149 | 0.126 | 0.179 |
| | Mean error§ | 20 | 0.226 | 0.197 | 0.185 | 0.158 | 0.177 | 0.185 | 0.158 |
| | | 40 | 0.296 | 0.234 | 0.187 | 0.150 | 0.192 | 0.190 | 0.150 |
| | | 60 | 0.464 | 0.216 | 0.204 | 0.158 | 0.210 | 0.205 | 0.157 |
| | | 80 | 1.248 | 0.288 | 0.265 | 0.180 | 0.272 | 0.267 | 0.181 |
| | Coverage | 20 | 0.939 | 0.964 | 0.985 | 0.992 | 0.982 | 0.980 | 0.992 |
| | | 40 | 0.957 | 0.981 | 0.996 | 0.998 | 0.990 | 0.997 | 0.998 |
| | | 60 | 0.959 | 0.997 | 1.000 | 1.000 | 1.000 | 1.000 | 1.000 |
| | | 80 | 0.990 | 1.000 | 1.000 | 1.000 | 1.000 | 1.000 | 1.000 |
| | Power | 20 | 0.722 | 0.885 | 0.801 | 0.916 | 0.822 | 0.808 | 0.916 |
| | | 40 | 0.466 | 0.836 | 0.678 | 0.935 | 0.672 | 0.670 | 0.940 |
| | | 60 | 0.217 | 0.500 | 0.353 | 0.942 | 0.376 | 0.381 | 0.959 |
| | | 80 | 0.048 | 0.114 | 0.040 | 0.896 | 0.050 | 0.043 | 0.891 |
| MNAR | Mean bias§ | 20 | -0.001 | 0.090 | 0.038 | 0.067 | 0.044 | 0.038 | 0.066 |
| | | 40 | -0.003 | 0.169 | 0.078 | 0.121 | 0.076 | 0.075 | 0.115 |
| | | 60 | -0.024 | 0.168 | 0.110 | 0.149 | 0.112 | 0.111 | 0.153 |
| | | 80 | 0.003 | 0.237 | 0.203 | 0.177 | 0.201 | 0.179 | 0.184 |
| | Mean error§ | 20 | 0.238 | 0.198 | 0.193 | 0.163 | 0.188 | 0.191 | 0.161 |
| | | 40 | 0.419 | 0.276 | 0.243 | 0.177 | 0.227 | 0.243 | 0.172 |
| | | 60 | 0.518 | 0.245 | 0.218 | 0.162 | 0.231 | 0.218 | 0.164 |
| | | 80 | 1.413 | 0.409 | 0.421 | 0.201 | 0.431 | 0.437 | 0.203 |
| | Coverage | 20 | 0.936 | 0.967 | 0.977 | 0.994 | 0.981 | 0.983 | 0.991 |
| | | 40 | 0.941 | 0.990 | 0.996 | 1.000 | 0.996 | 0.994 | 1.000 |
| | | 60 | 0.966 | 0.999 | 1.000 | 1.000 | 1.000 | 1.000 | 1.000 |
| | | 80 | 0.992 | 1.000 | 1.000 | 1.000 | 1.000 | 1.000 | 1.000 |
| | Power | 20 | 0.675 | 0.831 | 0.773 | 0.867 | 0.780 | 0.765 | 0.881 |
| | | 40 | 0.323 | 0.535 | 0.396 | 0.693 | 0.393 | 0.399 | 0.672 |
| | | 60 | 0.173 | 0.388 | 0.264 | 0.828 | 0.284 | 0.269 | 0.864 |
| | | 80 | 0.023 | 0.009 | 0.000 | 0.169 | 0.002 | 0.006 | 0.202 |

*Analysis model A: complete case analysis (no multiple imputation [mi]); B: no outcome imputation, not included in mi model; C: no outcome imputation, outcome imputed in mi model; D: outcome imputed and included in mi model; E: outcome imputed and included in mi model but then observations where it was imputed are deleted; F as in C but also including a second correlated outcome in the mi model; G as in D but also including a second correlated outcome in the mi model.

† Analysis model H does not include the covariate and hence not reported.

‡ Main models of interest, other models provided for comparison purposes.

§ Reported on log-odds scale and based on a true effect of log(1.2)

**Table S3: Performance results for covariate X, datasets of 10,000 observations*†**

| | | % miss | A | B | C‡ | D‡ | E‡ | F‡ | G‡ |
|---|---|---|---|---|---|---|---|---|---|
| MCAR | Mean bias§ | 20 | -0.003 | 0.081 | -0.026 | -0.045 | -0.026 | -0.026 | -0.045 |
| | | 40 | -0.002 | 0.164 | -0.049 | -0.077 | -0.049 | -0.050 | -0.075 |
| | | 60 | 0.001 | 0.248 | -0.069 | -0.098 | -0.073 | -0.068 | -0.096 |
| | | 80 | -0.040 | 0.318 | -0.116 | -0.118 | -0.101 | -0.112 | -0.117 |
| | Mean error§ | 20 | 0.056 | 0.086 | 0.054 | 0.058 | 0.053 | 0.054 | 0.059 |
| | | 40 | 0.078 | 0.164 | 0.071 | 0.083 | 0.071 | 0.072 | 0.082 |
| | | 60 | 0.111 | 0.248 | 0.095 | 0.108 | 0.099 | 0.095 | 0.107 |
| | | 80 | 0.233 | 0.318 | 0.170 | 0.164 | 0.163 | 0.167 | 0.163 |
| | Coverage | 20 | 0.945 | 0.815 | 0.955 | 0.932 | 0.958 | 0.954 | 0.923 |
| | | 40 | 0.941 | 0.500 | 0.951 | 0.888 | 0.943 | 0.947 | 0.890 |
| | | 60 | 0.955 | 0.318 | 0.964 | 0.883 | 0.949 | 0.960 | 0.870 |
| | | 80 | 0.941 | 0.511 | 0.955 | 0.884 | 0.960 | 0.950 | 0.881 |
| | Power | 20 | 1.000 | 1.000 | 1.000 | 1.000 | 1.000 | 1.000 | 1.000 |
| | | 40 | 1.000 | 1.000 | 1.000 | 1.000 | 1.000 | 1.000 | 1.000 |
| | | 60 | 0.995 | 1.000 | 0.999 | 1.000 | 0.999 | 0.999 | 1.000 |
| | | 80 | 0.631 | 1.000 | 0.778 | 0.889 | 0.809 | 0.790 | 0.905 |
| MAR | Mean bias§ | 20 | -0.004 | 0.060 | -0.020 | -0.034 | -0.019 | -0.020 | -0.033 |
| | | 40 | 0.002 | 0.126 | -0.026 | -0.045 | -0.026 | -0.025 | -0.043 |
| | | 60 | -0.001 | 0.229 | -0.056 | -0.081 | -0.059 | -0.057 | -0.079 |
| | | 80 | -0.017 | 0.301 | -0.059 | -0.065 | -0.052 | -0.057 | -0.061 |
| | Mean error§ | 20 | 0.054 | 0.069 | 0.052 | 0.054 | 0.048 | 0.052 | 0.053 |
| | | 40 | 0.061 | 0.126 | 0.058 | 0.062 | 0.060 | 0.058 | 0.062 |
| | | 60 | 0.105 | 0.229 | 0.092 | 0.099 | 0.088 | 0.092 | 0.099 |
| | | 80 | 0.182 | 0.301 | 0.153 | 0.145 | 0.149 | 0.151 | 0.144 |
| | Coverage | 20 | 0.939 | 0.873 | 0.946 | 0.934 | 0.965 | 0.948 | 0.936 |
| | | 40 | 0.970 | 0.655 | 0.970 | 0.944 | 0.957 | 0.971 | 0.941 |
| | | 60 | 0.944 | 0.340 | 0.952 | 0.873 | 0.957 | 0.952 | 0.900 |
| | | 80 | 0.949 | 0.398 | 0.953 | 0.924 | 0.952 | 0.947 | 0.928 |
| | Power | 20 | 1.000 | 1.000 | 1.000 | 1.000 | 1.000 | 1.000 | 1.000 |
| | | 40 | 1.000 | 1.000 | 1.000 | 1.000 | 1.000 | 1.000 | 1.000 |
| | | 60 | 1.000 | 1.000 | 0.999 | 1.000 | 1.000 | 1.000 | 1.000 |
| | | 80 | 0.843 | 1.000 | 0.891 | 0.924 | 0.901 | 0.883 | 0.930 |
| MNAR | Mean bias§ | 20 | -0.002 | 0.076 | -0.026 | -0.045 | -0.025 | -0.025 | -0.046 |
| | | 40 | -0.001 | 0.154 | -0.049 | -0.084 | -0.052 | -0.048 | -0.086 |
| | | 60 | 0.000 | 0.242 | -0.084 | -0.129 | -0.098 | -0.083 | -0.128 |
| | | 80 | 0.006 | 0.304 | -0.055 | -0.077 | -0.049 | -0.058 | -0.069 |
| | Mean error§ | 20 | 0.060 | 0.082 | 0.058 | 0.061 | 0.053 | 0.058 | 0.062 |
| | | 40 | 0.092 | 0.156 | 0.087 | 0.100 | 0.088 | 0.085 | 0.101 |
| | | 60 | 0.115 | 0.242 | 0.108 | 0.138 | 0.121 | 0.108 | 0.136 |
| | | 80 | 0.308 | 0.304 | 0.240 | 0.229 | 0.252 | 0.238 | 0.226 |
| | Coverage | 20 | 0.950 | 0.849 | 0.959 | 0.946 | 0.972 | 0.958 | 0.941 |
| | | 40 | 0.947 | 0.770 | 0.965 | 0.923 | 0.955 | 0.965 | 0.919 |
| | | 60 | 0.965 | 0.456 | 0.942 | 0.822 | 0.925 | 0.947 | 0.800 |
| | | 80 | 0.964 | 0.960 | 0.964 | 0.951 | 0.960 | 0.965 | 0.935 |
| | Power | 20 | 1.000 | 1.000 | 1.000 | 1.000 | 1.000 | 1.000 | 1.000 |
| | | 40 | 1.000 | 1.000 | 1.000 | 1.000 | 0.999 | 1.000 | 1.000 |
| | | 60 | 0.996 | 1.000 | 0.999 | 0.999 | 0.996 | 0.997 | 0.999 |
| | | 80 | 0.412 | 0.999 | 0.439 | 0.546 | 0.472 | 0.456 | 0.574 |

*Analysis model A: complete case analysis (no multiple imputation [mi]); B: no outcome imputation, not included in mi model; C: no outcome imputation, outcome imputed in mi model; D: outcome imputed and included in mi model; E: outcome imputed and included in mi model but then observations where it was imputed are deleted; F as in C but also including a second correlated outcome in the mi model; G as in D but also including a second correlated outcome in the mi model.

† Analysis model H does not include the covariate and hence not reported.

‡ Main models of interest, other models provided for comparison purposes.

§ Reported on log-odds scale and based on a true effect of log(1.5)

**Table S4: Performance results for interaction term E*X, datasets of 10,000 observations*†**

| | | % miss | A | B | C‡ | D‡ | E‡ | F‡ | G‡ |
|---|---|---|---|---|---|---|---|---|---|
| MCAR | Mean bias§ | 20 | 0.001 | 0.043 | 0.038 | 0.066 | 0.037 | 0.037 | 0.066 |
| | | 40 | 0.002 | 0.081 | 0.073 | 0.118 | 0.073 | 0.076 | 0.115 |
| | | 60 | 0.000 | 0.115 | 0.110 | 0.154 | 0.107 | 0.109 | 0.152 |
| | | 80 | 0.027 | 0.157 | 0.152 | 0.175 | 0.144 | 0.152 | 0.176 |
| | Mean error§ | 20 | 0.069 | 0.066 | 0.064 | 0.074 | 0.063 | 0.065 | 0.074 |
| | | 40 | 0.096 | 0.090 | 0.085 | 0.118 | 0.082 | 0.087 | 0.115 |
| | | 60 | 0.135 | 0.118 | 0.113 | 0.154 | 0.112 | 0.113 | 0.152 |
| | | 80 | 0.285 | 0.159 | 0.154 | 0.175 | 0.148 | 0.154 | 0.176 |
| | Coverage | 20 | 0.940 | 0.955 | 0.966 | 0.948 | 0.964 | 0.961 | 0.944 |
| | | 40 | 0.941 | 0.956 | 0.963 | 0.866 | 0.965 | 0.953 | 0.878 |
| | | 60 | 0.960 | 0.970 | 0.987 | 0.757 | 0.987 | 0.986 | 0.784 |
| | | 80 | 0.943 | 0.996 | 0.999 | 0.695 | 1.000 | 0.998 | 0.648 |
| | Power | 20 | 1.000 | 1.000 | 1.000 | 1.000 | 1.000 | 1.000 | 1.000 |
| | | 40 | 1.000 | 1.000 | 1.000 | 1.000 | 1.000 | 1.000 | 1.000 |
| | | 60 | 0.988 | 1.000 | 1.000 | 1.000 | 1.000 | 1.000 | 1.000 |
| | | 80 | 0.558 | 1.000 | 1.000 | 1.000 | 1.000 | 1.000 | 1.000 |
| MAR | Mean bias§ | 20 | 0.001 | 0.095 | 0.035 | 0.063 | 0.035 | 0.036 | 0.063 |
| | | 40 | -0.006 | 0.178 | 0.072 | 0.114 | 0.073 | 0.070 | 0.111 |
| | | 60 | -0.001 | 0.158 | 0.109 | 0.152 | 0.112 | 0.109 | 0.151 |
| | | 80 | -0.010 | 0.210 | 0.150 | 0.176 | 0.146 | 0.146 | 0.176 |
| | Mean error§ | 20 | 0.070 | 0.101 | 0.064 | 0.071 | 0.061 | 0.064 | 0.071 |
| | | 40 | 0.087 | 0.178 | 0.081 | 0.114 | 0.084 | 0.080 | 0.112 |
| | | 60 | 0.131 | 0.158 | 0.113 | 0.152 | 0.115 | 0.113 | 0.151 |
| | | 80 | 0.292 | 0.210 | 0.152 | 0.176 | 0.148 | 0.148 | 0.176 |
| | Coverage | 20 | 0.935 | 0.817 | 0.952 | 0.930 | 0.963 | 0.951 | 0.936 |
| | | 40 | 0.957 | 0.597 | 0.971 | 0.885 | 0.959 | 0.973 | 0.893 |
| | | 60 | 0.951 | 0.913 | 0.983 | 0.795 | 0.979 | 0.987 | 0.789 |
| | | 80 | 0.951 | 0.981 | 0.999 | 0.640 | 0.998 | 0.998 | 0.627 |
| | Power | 20 | 1.000 | 1.000 | 1.000 | 1.000 | 1.000 | 1.000 | 1.000 |
| | | 40 | 1.000 | 1.000 | 1.000 | 1.000 | 1.000 | 1.000 | 1.000 |
| | | 60 | 0.981 | 1.000 | 1.000 | 1.000 | 1.000 | 1.000 | 1.000 |
| | | 80 | 0.497 | 1.000 | 1.000 | 1.000 | 1.000 | 1.000 | 1.000 |
| MNAR | Mean bias§ | 20 | 0.001 | 0.088 | 0.040 | 0.068 | 0.037 | 0.039 | 0.065 |
| | | 40 | 0.000 | 0.169 | 0.080 | 0.118 | 0.072 | 0.080 | 0.116 |
| | | 60 | -0.008 | 0.165 | 0.111 | 0.151 | 0.111 | 0.110 | 0.151 |
| | | 80 | -0.009 | 0.222 | 0.148 | 0.176 | 0.147 | 0.149 | 0.175 |
| | Mean error§ | 20 | 0.074 | 0.097 | 0.068 | 0.077 | 0.064 | 0.068 | 0.074 |
| | | 40 | 0.115 | 0.170 | 0.095 | 0.119 | 0.090 | 0.095 | 0.116 |
| | | 60 | 0.149 | 0.166 | 0.115 | 0.151 | 0.115 | 0.115 | 0.151 |
| | | 80 | 0.453 | 0.227 | 0.164 | 0.176 | 0.163 | 0.164 | 0.175 |
| | Coverage | 20 | 0.933 | 0.878 | 0.959 | 0.940 | 0.974 | 0.960 | 0.949 |
| | | 40 | 0.959 | 0.832 | 0.983 | 0.957 | 0.981 | 0.982 | 0.959 |
| | | 60 | 0.952 | 0.933 | 0.987 | 0.863 | 0.991 | 0.990 | 0.853 |
| | | 80 | 0.957 | 0.998 | 1.000 | 0.987 | 1.000 | 1.000 | 0.993 |
| | Power | 20 | 1.000 | 1.000 | 1.000 | 1.000 | 1.000 | 1.000 | 1.000 |
| | | 40 | 0.997 | 1.000 | 1.000 | 1.000 | 1.000 | 1.000 | 1.000 |
| | | 60 | 0.947 | 1.000 | 1.000 | 1.000 | 1.000 | 1.000 | 1.000 |
| | | 80 | 0.217 | 0.965 | 0.883 | 1.000 | 0.879 | 0.902 | 1.000 |

*Analysis model A: complete case analysis (no multiple imputation [mi]); B: no outcome imputation, not included in mi model; C: no outcome imputation, outcome imputed in mi model; D: outcome imputed and included in mi model; E: outcome imputed and included in mi model but then observations where it was imputed are deleted; F as in C but also including a second correlated outcome in the mi model; G as in D but also including a second correlated outcome in the mi model.
† Analysis model H does not include the covariate and hence not reported.
‡ Main models of interest, other models provided for comparison purposes.
§ Reported on log-odds scale and based on a true effect of log(1.2)

**Table S5: Successful convergences in 1000 simulations***

| 1000 observations | | | | | | | | | |
|---|---|---|---|---|---|---|---|---|---|
| | % miss | A | B | C† | D† | E† | F† | G† | H |
| MCAR | 20% | 1000 | 1000 | 1000 | 1000 | 1000 | 1000 | 1000 | 1000 |
| | 40% | 1000 | 1000 | 1000 | 1000 | 1000 | 1000 | 1000 | 1000 |
| | 60% | 999 | 1000 | 1000 | 1000 | 1000 | 1000 | 1000 | 1000 |
| | 80% | 751 | 997 | 993 | 997 | 999 | 866 | 997 | 997 |
| MAR | 20% | 1000 | 1000 | 1000 | 1000 | 1000 | 1000 | 1000 | 1000 |
| | 40% | 1000 | 1000 | 1000 | 1000 | 1000 | 1000 | 1000 | 1000 |
| | 60% | 999 | 1000 | 1000 | 1000 | 1000 | 1000 | 1000 | 1000 |
| | 80% | 714 | 997 | 991 | 997 | 998 | 938 | 997 | 997 |
| MNAR | 20% | 1000 | 1000 | 1000 | 1000 | 1000 | 1000 | 1000 | 1000 |
| | 40% | 1000 | 1000 | 1000 | 1000 | 1000 | 1000 | 1000 | 1000 |
| | 60% | 996 | 1000 | 1000 | 1000 | 1000 | 1000 | 1000 | 1000 |
| | 80% | 397 | 968 | 887 | 961 | 943 | 855 | 962 | 982 |
| 10000 observations | | | | | | | | | |
| | % miss | A | B | C† | D† | E† | F† | G† | H |
| MCAR | 20% | 1000 | 1000 | 1000 | 1000 | 1000 | 1000 | 1000 | 1000 |
| | 40% | 1000 | 1000 | 1000 | 1000 | 1000 | 1000 | 1000 | 1000 |
| | 60% | 1000 | 1000 | 1000 | 1000 | 1000 | 1000 | 1000 | 1000 |
| | 80% | 1000 | 1000 | 1000 | 1000 | 1000 | 1000 | 1000 | 1000 |
| MAR | 20% | 1000 | 1000 | 1000 | 1000 | 1000 | 1000 | 1000 | 1000 |
| | 40% | 1000 | 1000 | 1000 | 1000 | 1000 | 1000 | 1000 | 1000 |
| | 60% | 1000 | 1000 | 1000 | 1000 | 1000 | 1000 | 1000 | 1000 |
| | 80% | 1000 | 1000 | 1000 | 1000 | 1000 | 1000 | 1000 | 1000 |
| MNAR | 20% | 1000 | 1000 | 1000 | 1000 | 1000 | 1000 | 1000 | 1000 |
| | 40% | 1000 | 1000 | 1000 | 1000 | 1000 | 1000 | 1000 | 1000 |
| | 60% | 1000 | 1000 | 1000 | 1000 | 1000 | 1000 | 1000 | 1000 |
| | 80% | 1000 | 1000 | 1000 | 1000 | 1000 | 1000 | 1000 | 1000 |

*Analysis model A: complete case analysis (no multiple imputation [mi]); B: no outcome imputation, not included in mi model; C: no outcome imputation, outcome imputed in mi model; D: outcome imputed and included in mi model; E: outcome imputed and included in mi model but then observations where it was imputed are deleted; F as in C but also including a second correlated outcome in the mi model; G as in D but also including a second correlated outcome in the mi model; H as in D but the mi and analysis models do not include the covariate.
† Main models of interest, other models provided for comparison purposes.

**Figure S1: Mean Bias (top) and Mean absolute Error (bottom) for exposure E in datasets of 1000 observations, continuous outcome**

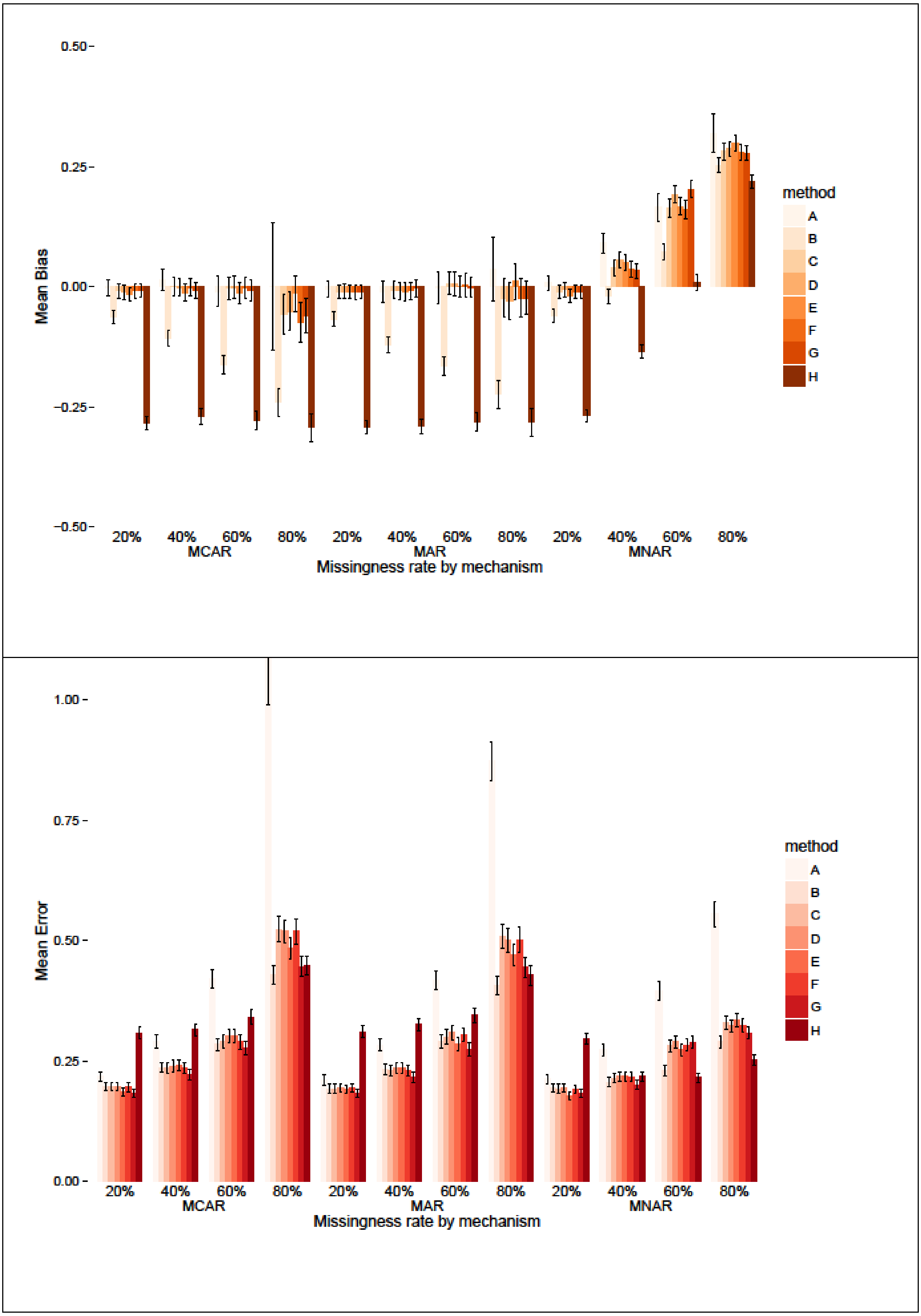

**Figure S2: Coverage (top) and Power (bottom) for exposure E in datasets of 1000 observations, continuous outcome**

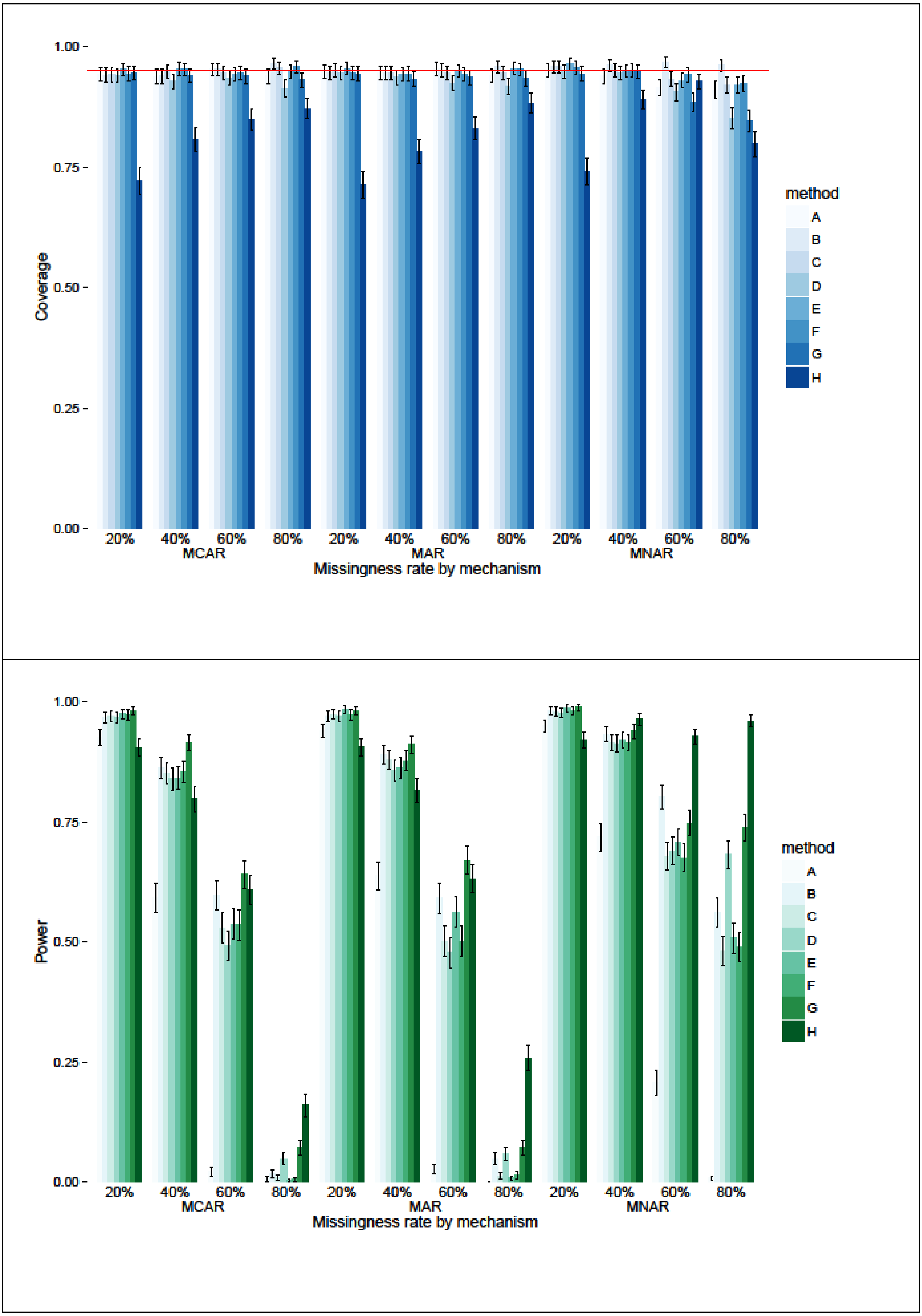

Supplement: Additional file 2: — Supplementary file for “Outcome-sensitive Multiple Imputation: a Simulation Study”. Additional results for the covariate and the interaction term for the main analyses, but also all results from sensitivity analyses. (PDF 480 kb) [file 12874_2016_281_MOESM2_ESM.pdf]
